# Hypnotism
1Read at a meeting of the Bath and Bristol Branch of the British Medical Association, November 25th, 1909.


**Published:** 1910-03

**Authors:** Hugh P. Costobadie


### HYPNOTISM.[1]

BY

HUGH P. COSTOBADIE, M.R.C.S., L.R.C.P.

---

On October 18th, 1909, Dr. George H. Savage delivered the Harveian Oration on Experimental Psychology and Hypnotism. In 1846—sixty-three years ago—Dr. John Elliotson also delivered the Harveian Oration of that year on the same subject, under the name of Mesmerism. The earlier lecturer was totally disregarded.

The striking point about these two lectures, so widely separated by time, is that neither speaker asks more of his hearers than that they should examine thoroughly and weigh carefully the usefulness or otherwise of hypnotism in medicine; and appropriately enough, each endeavours to bring his point home to his hearers by the same means, that is, by using Harvey's own words.

Bramwell tells us in his short history of John Elliotson that these words occurred in his oration: "We should always have before our eyes that memorable passage in Harvey's works, 'True philosophers, compelled by the love of truth and wisdom, never fancy themselves so wise and full of sense as not to yield

[1] Read at a meeting of the Bath and Bristol Branch of the British Medical Association, November 25th, 1909.

to truth from any source and at all times; nor are they so narrow-minded as to believe that any art or science has been handed down in such a state of perfection to us by our predecessors that nothing remains for future history.'" And Dr. Savage concludes with Harvey's words, 'I therefore whisper in your ear, friendly hearer, and recommend you to weigh carefully in the balance the exact expression of all that I have delivered in this my exercise."

John Elliotson, whatever may have been his personal shortcomings, was wrecked professionally by his belief in hypnotism; but such narrow-minded persecution as he suffered is to-day impossible amongst men who are grown used to meet great changes calmly and critically, and who, while still strongly conservative, are to-day possessed of vastly greater individuality

Now, what does hypnotism do to make itself useful in the study and treatment of disease? The answer to the question is, that it increases suggestibility.

Everyone is to a greater or less extent susceptible to suggestion, and suggestion is, in Moll's words, "a method of producing an effect by calling imagination into play." For example: You know, as do most people, that if a stick of sealing-wax be rubbed with a dry silk handkerchief it will pick up a small fragment of paper. Three sticks of sealing-wax are placed on a table before several persons, and they are told that an electric shock can be obtained from any one of them by touching it after it has been well rubbed. One of the onlookers is sent out of the room after it has been explained to him that during his absence one of the sticks will be well rubbed; that when he returns he is to touch them lightly with his finger, and by means of the shock given off by one of them he will be able to distinguish with certainty the stick that has been so treated. After he is gone, the remaining persons are told to agree with him in whichever stick he selects, but that none of the pieces will be rubbed, as in fact that would be useless. The subject of the experiment on his return will always indicate a stick as being one that gives a slight tingling to his finger; and if the experiment be repeated, after it has been impressed upon him that to give a greater shock a more vigorous rubbing will this time be given to one of the sticks during his absence, he will make his next selection with increased assurance, and by a good subject the extent and character of the sensation received is sometimes voluntarily described with surprising detail.

So greatly, however, is suggestibility increased by hypnotism, that it is only necessary to tell a patient in a deep hypnotic sleep that he has just received an electric shock for his muscles to tighten and his face to twitch in a way which shows without doubt that he believes he feels it, and moreover he will tell you that he does. Now, every sick person is subjected, knowingly or unknowingly by his doctor, to treatment by suggestion.

You are all aware of the fact that the thoroughness or the carelessness of the examination, the hopefulness or hopelessness and so on of the physician, influences the patient for good or evil, and oftentimes does more than the medicine prescribed in effecting a cure ; so that you will agree that it is this increase of suggestibility produced by the hypnosis that makes it such a very definitely useful though limited adjunct to therapeutics.

What the limits may be I think no one knows, for they largely depend upon an individual operator and a particular patient: I mean that one operator might cure a particular patient of, say, dysmenorrhœa, where another operator with the same patient would fail, or the same operator with another patient would be also unsuccessful.

Different authors have given lists of conditions in which they have found hypnotism useful, and quote many cases in proof. On analysis, it is seen that those in which the best results have been obtained are those which are found so difficult to deal with by the more ordinary methods which we have at hand ; I mean that large class called by us functional diseases—tics, tremors, and nervous spasms and cramps, and some neuralgic pains and headaches, some hysterical paralyses, convulsions and aphonia, many phobias and so on, while sleeplessness and sleep-walking can be cured. I have had cases of hysterical twitchings and tremors, of nocturnal enuresis, of neuralgias, of headaches, and of sleeplessness which I have been able to cure, and others of chronic constipation, of dyspeptic discomfort, and a case of the morning vomiting of pregnancy. I have been able to benefit for a few months a case of melancholia ; and I once tried its use in labour, but I was unable to control the pain ; and in a case of epilepsy it had no good result in my hands.

Sir Francis Cruise once told me of a most useful case which he had cured.

The patient was a dentist, who whenever he took up an instrument to operate, but at no other time, was troubled with a coarse tremor of the hand and arm, which rendered his work most difficult and after a time impossible. Sir Francis Cruise saw him, and told him that he could be cured, and by means of suggestion during hypnosis the tremor was made to disappear after a few sittings. Such a complaint to a dentist would totally incapacitate him from work, and I know of no other method of treatment which would have cured him either so quickly, or without taking him away from his duties, or by means of which so successful a termination could have been so definitely predicted.

I will give shortly one more interesting illustration of its usefulness from among my own cases. On November 4th of last year W. G., a miner, twenty-three years of age, and who comes of a good, hard-working family, came to see me because he was afraid to return to his work down the mine. Eleven weeks previously, when "getting coal," he had crushed his hand, and although this had healed in three weeks, he had not since been able to persuade himself to return to his labour, though he often got up in the morning at the proper hour and put on his working clothes, only to take them off again, as his fear of going underground overcame his determination to work. He had already consulted about his present trouble four separate medical men, who had all, he told me, carefully examined his heart, nervous system, urine, etc., and had ordered him treatment and change of air, but without result.

He was depressed and anxious about himself. His pupils were unstable and his pulse-rate a little increased, but I could discover nothing further amiss, and he had been gaining in weight. I assured him, as his other doctors had done, that he was physically fit and capable of doing the hardest of work. I then hypnotised him, and repeated my assurance, and told him that his fears were ill-founded. I suggested that he should return to his work on the morning of the second day, that is to say November 6th. The next day, November 5th, I repeated the suggestion during hypnosis, and the following morning he returned to work, reporting himself six days later as being perfectly well, and he has remained at work ever since.

Alcoholism and drug habits can be cured by this means, and it is most useful in acute conditions in relieving disagreeable symptoms and giving sleep, and I have myself used it to relieve the myalgia of tonsillitis and the restlessness and headache of influenza. I have once or twice used hypnosis when my partner, Dr. Pollard, has performed some slight operation, such as a dental extraction, and the patient has felt no pain.

But lists of diseases in which it has been used with more or less success can be found in any one of the many books on hypnotism, so I will not trouble you further with this part of the subject, but will now try to show how it may be used in experimental psychology, with a possible chance of putting us on the right road to discovering something of the mystery of the working of the brain. Before doing so, however, I want to say a word about the dangers of hypnotism.

Of undue influence and criminal actions almost all writers show that, whilst it is under certain exceptional circumstances possible, the cases are few, and that chloroform put to the same use would be a safer and surer means to employ, as would certainly be known to the criminal hypnotist, who would realise the possibilities of abruptly arousing his victim and the uncertainty of the completeness of the loss of memory, or the period over which it would extend. The popular idea is, as is so often the case, exaggerated and distorted.

Of the complications following hypnosis, I have had headache following the first sitting on more than one occasion, but it has never persisted after I have made suitable suggestions on re-hypnotising the patient. Again, I have once or twice had patients complain of coldness of the extremities ; but this has also been easy to deal with. A more serious condition was a tremor of the right arm, which came on while a patient was in an hypnotic sleep, and which, although I arrested it almost immediately, recurred as soon as she was awakened, and it was only after a prolonged sleep that I was finally able to rid her of it. This I traced to the fact that she was lying on a couch with a soft cushion beneath her head and shoulders, and under which a large, hard book had been placed, of which I was unaware. This was under the patient's right shoulder, and although I asked her directly she was asleep if she was comfortable, it was not until after the tremor had commenced, and I was questioning her as to its cause, that she told me that something hard beneath the cushion was giving her considerable discomfort.

I think that it was this continuous pressure that during her sleep centred her attention on her arm, and produced the tremor by auto-suggestion.

By other operators I have twice been told that they have had patients who were troubled by headache produced by hypnosis, and another doctor told me of an attack of hysteria in a patient whom he had hypnotised and left for a moment, and who was in his absence abruptly awakened by some of the other patients in the ward pinching and smacking her.

In the literature on the subject there have been reported few, if any, cases of serious trouble following the use of hypnotism by skilled operators, although in unskilled hands serious and permanent damage may be done.

One of the most marvellous phenomena of hypnotism is post-hypnotic suggestion.

If a patient when hypnotised be told that in ten minutes after waking he will take out his handkerchief and tie a knot in it he will do so, and in the same way he will on waking see a picture on a blank sheet of paper if this has been suggested while in hypnosis. It is to the nature of this picture that I wish now to draw your attention. The following experiment, which has been published under the initials H. E., whom I know personally, and whose original experiments I have repeated and tested, will well serve my purpose. Such a picture belonging to the class I have named was seen by X., who, before it faded, described minutely a fine, well-furnished room, containing amongst other things a clock unusually large. Now, on being asked where he had seen such a room, he was certain that he had never seen one like it, " for if I had I should have remembered it by the curious clock."

On being hypnotised and asked the same question, he replied at once, " At so-and-so ; the clock cost hundreds of pounds."

On being awakened, he was told that he had seen such a room, but he was still unable to recall it until told where, when he readily agreed, and added that it was five years ago.

Now, this is evidently an example of what is called sub-conscious memory ; but the still more interesting point is what made X., who believed he had never thought of the room since the day he saw it, recall it at the particular moment when he was looking at that blank sheet of paper ?

He himself could give no reason at all, but on being re-hypnotised he said, " The gas thing in the middle of the room was all made of glass—it was beautiful. The little knobs on the chandeliers in the church to-day " (he had been to look over an old church earlier in the day, and had examined, standing on a chair, the cut-glass knobs on the church lamps) " were made like it, and I suppose that made me think of the thing again." On being wakened, he did not remember thinking of the room when looking at the candles in the church.

Now, probably the greatest stimulus given, say, within that day to his sub-conscious memory was by means of those cut-glass knobs, and the result had not yet faded when he looked at the paper, and so the production of this particular picture came about.

You will see first, then, that the subject had apparently forgotten the room, and next that he could not, even after he had recalled it, remember the cause of its coming back to his memory, but that such a cause did exist the experiment proves. This was the last arch in the bridge of memory from the time of his seeing the room to the time of the experiment, and we may take it that these arches are separated from each other by gradually-increasing periods of time, for he would undoubtedly at first in his waking state have remembered from day to day, then from week to week, and so on.

The memory from any given point is kept alive by these arches, at some time changed into the region of sub-consciousness.

Of course, conscious memory may recur at any time; but from the case quoted, I would suggest that this had only taken place at some period long previously. I think we can also deduce this fact, that sub-conscious memory, if it be not actually indestructible, is "good" over a longer period than conscious memory, and only requires an appropriate suggestion to lift it into the region of consciousness.

The sub-conscious memory may, therefore, be an important factor in disease; for may not the repeated stimulus of sub-conscious memory, acting, for example, on imagination, be the obscure origin of some of those still more obscure conditions which occur in so many functional and mental diseases, if not the cause of some of those diseases themselves?

But this is a bare indication of a line of experiment that is well worth further investigation.

The subject I have dealt with is of vast extent, and my paper of necessity contracted, but I trust you may find in it some matter worthy of debate.

